# Opinion controversy to chromium picolinate therapy’s safety and efficacy: ignoring ‘anecdotes’ of case reports or recognising individual risks and new guidelines urgency to introduce innovation by predictive diagnostics?

**DOI:** 10.1186/1878-5085-3-11

**Published:** 2012-10-07

**Authors:** Olga Golubnitschaja, Kristina Yeghiazaryan

**Affiliations:** 1 Department of Radiology, Rheinische Friedrich-Wilhelms-University of Bonn, Sigmund-Freud-Str. 25, Bonn, 53105, Germany

**Keywords:** Patient records, Individual profiles, Subcellular imaging patterns, Therapy response, Pre/diabetes care, New guidelines, Predictive preventive personalised medicine, Dietary supplements, Healthcare, Integrative medicine

## Abstract

Due to the important physiologic function of trivalent chromium in glucose/insulin homeostasis, some commercial organisations promote Cr^3+^ supplements in maintaining proper carbohydrate and lipid metabolism; regulation of reducing carbohydrate carvings and appetite; prevention of insulin resistance and glucose intolerance; regulation of body composition, including reducing fat mass and increasing lean body mass; optimal body building for athletes; losing weight; treatment of atypical depression as an antidepressant; and prevention of obesity and type 2 diabetes mellitus. On one hand, case reports are commented as ‘nonevidence-based anecdotes’. On the other hand, a number of independent studies warn against adverse health outcomes assigned to chromium picolinate (CrPic) dietary application. This review analyses opinion controversies, demonstrates highly individual reactions towards CrPic dietary supplements and highlights risks when the dietary supplements are used freely as therapeutic agents, without application of advanced diagnostic tools to predict individual outcomes.

## Review

### Changing long-held beliefs is never easy

**Figure 1:**
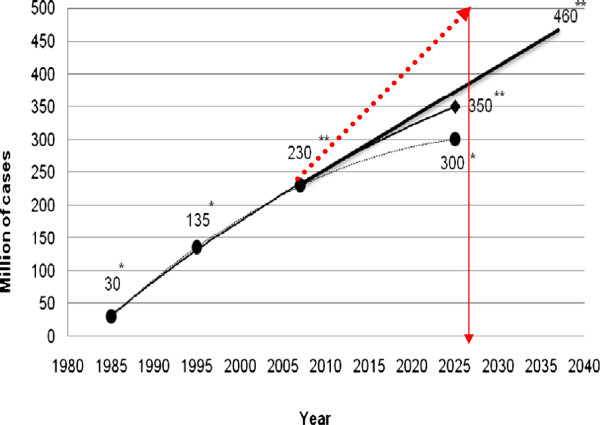
** Worldwide prognosis of the epidemic scale of diabetes. ***Single asterisks* denote estimations as published around the year 2000; *double asterisks* denote stepwise worsening prognosis (from *black circles* to *black diamond*) as published in 2003–2008; current prognoses are marked in *red colour* (adapted from [1,2]).

As a consequence of the accumulating clinical data and knowledge about the epidemiology and pathological mechanisms of the most frequent causes of morbidity and mortality, we are currently reconsidering our view of the origins and progression of cardiovascular, oncologic and neurodegenerative diseases. The majority of these pathologies are of chronic nature: they progress from precursor lesions over one or even several decades of life; therefore, it is often too late for effective therapeutic intervention. An excellent example is the epidemic scale of type 2 diabetes mellitus witnessed in the European Union. In most industrialised countries and countries with large populations, the permanently growing cohort of diabetics creates a serious healthcare problem and a dramatic health economic burden. The estimate for diabetes prevalence in the years 2025–2030 is half a billion patients worldwide (see Figure 1).

**Figure 2:**
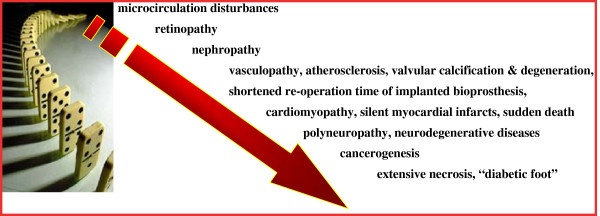
** Severe complications developed secondary to diabetes: from ‘upstream’ (*up*) to ‘downstream’ (*bottom*) in the cascade of pathologic processes (adapted from [**[4]**]).**

Moreover, the contemporary onset of the dominant type 2 diabetes was already observed in subpopulations of teenagers [3]. Severe complications secondary to early onset of diabetes mellitus, such as retinopathy, nephropathy, silent ischaemia, dementia and cancer (Figure 2), soon may lead to collapsing healthcare systems [4]. 

Optimistic *versus* pessimistic prognosis in future developments of the healthcare sector depends much on diagnostic, preventive and treatment approaches which diabetes care will preferably adopt in the near future. Without innovation in healthcare, diabetes-related complications may cause such a dramatic level of burden that any performance of personalised medicine will not be feasible from an economical point of view. In contrast, effective utilisation of advanced early/predictive diagnostics and targeted prevention could enable rapidly ageing populations in Europe and elsewhere to be economically effective for the society. Therefore, it is timely to consider the integrative medical approaches to advance pre/diabetes care utilising the innovation by predictive diagnostics as the basis for concomitant targeted prevention, patient profiling as the basis for individualised treatment algorithms and medical approaches tailored to the patient [5-8].

The message is that new guidelines should create a robust juristic and economic platform for advanced medical services utilising the cost-effective models of risk assessment followed by tailored treatments focussed on the precursor stages of chronic disease [9].

### Potential role of biologically active chromium in diabetes prevention

Chromium is an essential trace element in human physiology. The physiologic value of chromium compounds depends on the oxidative states of chromium ions that vary from bivalent to hexavalent ones. The best investigated forms are trivalent and hexavalent chromium compounds. The latter is frequently used in a variety of industrial technologies. Chronic exposure to it has been shown to be responsible for a spectrum of severe health problems affecting the skin and inner organs [10]. Investigations of its carcinogenic effects are in progress at molecular and cellular levels [11,12]. In contrast, trivalent chromium is considered physiologically safe and is an essential trace metal functionally involved in several metabolic pathways in human beings as published elsewhere. The recommended daily allowance of trivalent chromium corresponds to the range of 50–200 μg [13]. Dietary deficiency of chromium leads to disturbances in carbohydrate metabolisms; increases risk of glucose intolerance, insulin resistance, hyperglycaemia and predisposes to obesity and type 2 diabetes mellitus [14,15].

### Controversial benefits by Cr^3+^ nutrient supplement in foods

Biologically valuable forms of chromium are compounds of trivalent chromium bound to low molecular weight organic complexes such as oligopeptides. This recognition led to the development of a commercial branch with Cr^3+^ nutrient supplements which suppose to promote or even improve health status.

What are the currently available ‘pro’ *versus* ‘contra’ arguments to approve/disprove potential benefits of Cr^3+^ nutrient supplement?

Chromium is an essential metal, but only traces of trivalent chromium are utilised in the human body for glucose conversion by insulin-driven reactions in carbohydrate metabolic pathways. Furthermore, naturally available food sources used in well-balanced diets are capable to cover the requirement of trivalent chromium completely: fruits, vegetables, meat, fish, grains, brewer’s yeast, etc. [16]. Cr deficiency cases are extremely rare, and evidence-based biochemical justification for artificial source needed additionally to standard foods has not been provided in the literature. Nevertheless, due to the important physiologic function of trivalent chromium in maintaining proper carbohydrate and lipid metabolism and glucose/insulin homeostasis, some commercial organisations promote Cr^3+^ nutrient supplements in

regulation of reducing carbohydrate carvings and appetite

prevention of insulin resistance and glucose intolerance

regulation of body composition, including reducing fat mass and increasing lean body mass

optimal body building for athletes

losing weight

treatment of atypical depression as an antidepressant

prevention of obesity and type 2 diabetes mellitus.

The widely spread chemical compound used as a trivalent chromium nutrient supplement is chromium picolinate (CrPic). CrPic has become a very popular nutritional supplement for treating type 2 diabetic, obese and diabetes-predisposed individuals [17]. However, over a decade of human studies with CrPic indicate that the supplement has not demonstrated effects on the body composition of healthy individuals, even when taken in combination with an exercise training programme [18]. Also, a potential weight loss by CrPic therapy has not been confirmed [19].

After the evidence-based search into the issue, the US Food and Drug Administration concluded that the ‘relationship between CrPic intake and insulin resistance is highly uncertain’ [20]. Furthermore, some later studies have concluded that ‘CrPic does not improve key features of metabolic syndrome in obese nondiabetic adults’ [21]. Nevertheless, it is reported that CrPic generates sales for more than US$100 million annually [22]. The CrPic supplements are freely available in numerous forms including chewing gums, pills, sports drinks and nutrition bars [23]. CrPic is commonly supplied in the range of 200–500 μg as a daily dose [13]. A big advantage of CrPic is its high bioavailability due to increased absorbing capacity in human tissues compared with dietary chromium [24]. However, the mechanism of action of CrPic at molecular level is not completely understood. In 1999, Speetjens et al. reported that CrPic cleaves DNA molecules by a radical mechanism: in the presence of oxygen from air and biologically available reductants, CrPic generates hydroxyl radicals damaging DNA molecules [25]. Further, organisms exposed to CrPic demonstrate the tendency to accumulate intracellular Cr^3+^ ions that may lead to long-term genotoxic effects by formation of covalent bonds to DNA [26]. Taken together, cytotoxic, genotoxic and mutagenic effects as well as activity damaging to the mitochondria and induction of apoptosis have been reported for CrPic-using mammalian cell cultures, *Drosophila* and animal models [27-30]. Potential toxicity of CrPic has raised concerns about the safety of CrPic-related nutrient supplements.

### Animal experiments to model treatment effects of CrPic on human beings

**Figure 3:**
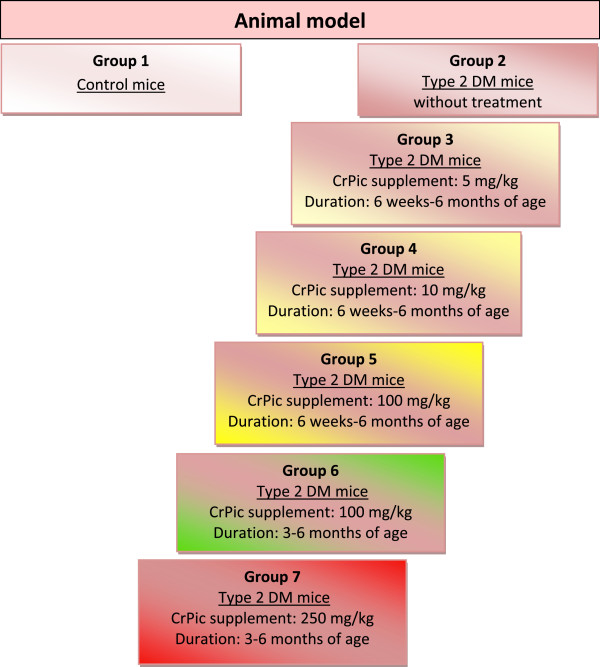
** Experimental design to model CrPic treatment effects.** Animals were arranged in eight groups. Group 1 represented the healthy control. Group 2 was the model mice for type 2 DM. Groups 3–7 were diabetes model mice treated under individual algorithms: 5 mg/kg CrPic from 6 weeks to 6 months of age (group 3), 10 mg/kg CrPic from 6 weeks to 6 months of age (group 4), 100 mg/kg CrPic from 6 weeks to 6 months of age (group 5), 100 mg/kg CrPic from 3 to 6 months of age (group 6), and 250 mg/kg CrPic from 3 to 6 months of age (group 7). Kidney tissue samples were collected and stored at −80 °C till the analysis described in the below section was performed.

To simulate treatment effects of CrPic, a spectrum of models has been used. To simulate type 2 diabetes mellitus (DM), a well-acknowledged animal model of db/db mice was utilised in a recent project [31]. The experimental design is summarised in Figure 3. 

### Subcellular imaging insights into CrPic therapy

#### Quantitative subcellular imaging by ‘comet assay’ analysis

The single-cell gel electrophoresis assay is a simple and effective method for evaluating DNA damage in cells. It is based on the ability of denatured, cleaved DNA fragments to migrate out of the cell under the influence of an electric field, whereas undamaged DNA remains within the confines of the nucleoid and migrates slower. The assessment of DNA damage is done via the evaluation of the DNA ‘comet’ tail shape and migration pattern. The cells are immobilised in a bed of low-melting point agarose, on a Trevigen CometSlide™ (Gaithersburg, MD, USA). After cell lysis, samples are treated with alkali to unwind and denature the DNA and hydrolyse sites of damage. After performing electrophoresis, staining with a fluorescent DNA-intercalating dye (SYBR® Green I; Trevigen Inc., Gaithersburg, MD, USA) is done, and the sample is visualised by epifluorescence microscopy. The alkaline electrophoresis is very sensitive and allows for the detection of small amounts of damage.

**Figure 4:**
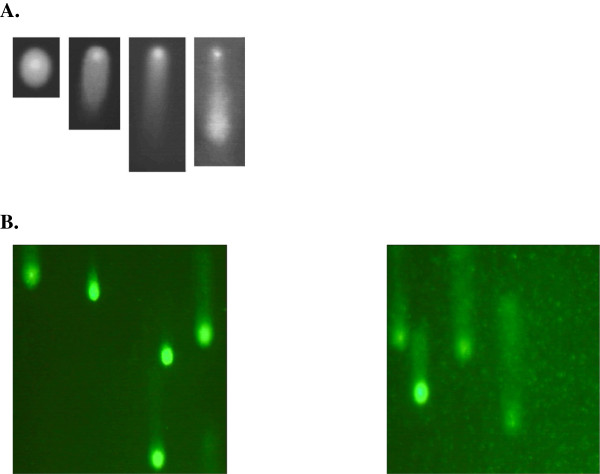
** Microscopic imaging.** (**A**) Comet classes I, II, III and IV (from *left* to *right*); (**B**) comet pattern typical for experimental group 1 (*left*, low apoptosis rates) and experimental groups 5–7 (*right*, high apoptosis rates).

In our experiments, DNA damage was designated to four classes based on the visual aspect considering the extent of DNA migration as published earlier [32]. Comets with a bright head and almost no tail were classified as class I, indicating minimal DNA damage, whereas comets with no visible head and a long diffuse tail were classified as class IV, revealing complete DNA fragmentation. Comets with intermediate characteristics were assigned to classes II and III based on the ratio *R* = *T*/*r*, in which *T* represents the comet’s tail length and *r* is the radius of the comet’s head. The characteristic value of *R* for class I is 1 and that for class IV is ∞ (*r* = 0). Comets with *R* values ranging between 1 < *R* > 3 were designated as class II. The comet classification is demonstrated in Figure 4A. Figure 4B demonstrates comet patterns typical for experimental group 1 (left) with the lowest level of comets of class IV (apoptosis) and experimental groups 5–7 (right) with the highest level of apoptosis. 

#### Statistical analysis reveals increased heterogeneity of individual reactions towards the highest doses of CrPic

**Figure 5:**
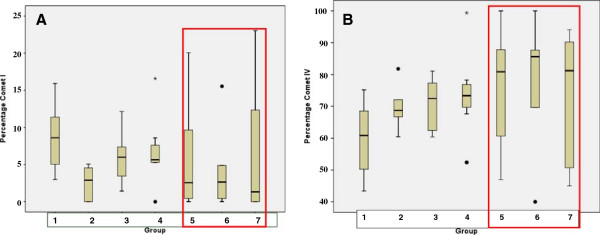
** Statistical analyses for the comet types (A) I and (B) IV (see ‘comet classification’ in Figure **4**A) in groups of comparison as described in Figure **3** (‘experimental design’).** Analyses were carried out using SPSS 17.0 software (SPSS, Chicago, IL, USA) by the application of univariable variance analysis with Bonferroni. The *red box* marks the groups with the highest deviation towards the mean value, i.e. the greatest heterogeneity within corresponding groups. The individual values which go overboard by statistical calculations are marked with *black circles* (for the standard deviation in groups 4 and 6) and *asterisk* (for the mean value in group 4).

Statistical analysis of comets in classes I (intact DNA) and IV (apoptosis) is demonstrated in Figure 5A, B, respectively. The red frame marks the experimental groups with the highest rates of statistical deviations towards the mean value. The highest heterogeneity is obvious for groups 5, 6 and 7, i.e. the experimental groups with the highest doses of CrPic applied as treatment.

##### DNA damage in untreated diabetic group is significantly higher compared to healthy control group

**Figure 6:**
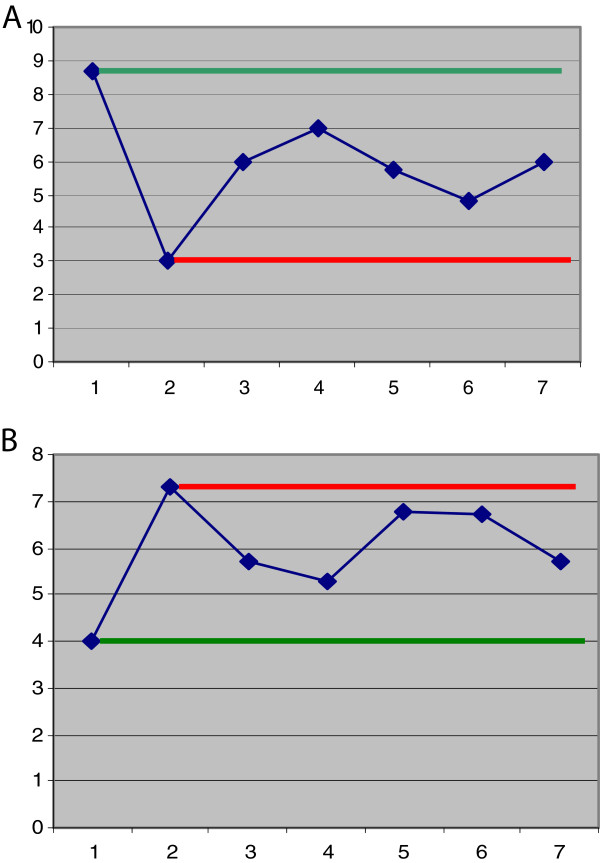
** Comparative analysis in the experimental groups.** (**A**) The diagram presents the mean values of the individual experimental group for the comets of class I (intact chromosomal DNA): *axe X*, group numbering (1–7); *axe Y*, corresponding mean values (% of comets). The highest and lowest rates correspond to control group 1 and untreated diabetic group 2, respectively. (**B**) The diagram presents the mean values of the individual experimental group for the ratio of comets with damaged DNA (classes III and IV) to the comets with undamaged DNA (classes I and II). Similarly to the diagram in (A), the highest and lowest rates of DNA damage correspond to control group 1 and untreated diabetic group 2, respectively. The *green lines* mark the level corresponding to the mean value of the control group; in contrast, the *red lines* mark the level corresponding to the mean value of the untreated diabetic group.

Quantitative subcellular imaging by comet assay analysis revealed significantly higher DNA damage in the untreated diabetic group compared to healthy controls (see Figure 6) that is well in agreement with data published and reviewed for diabetic patients [4,8]. 

##### Individuality within the untreated diabetic group

**Figure 7:**
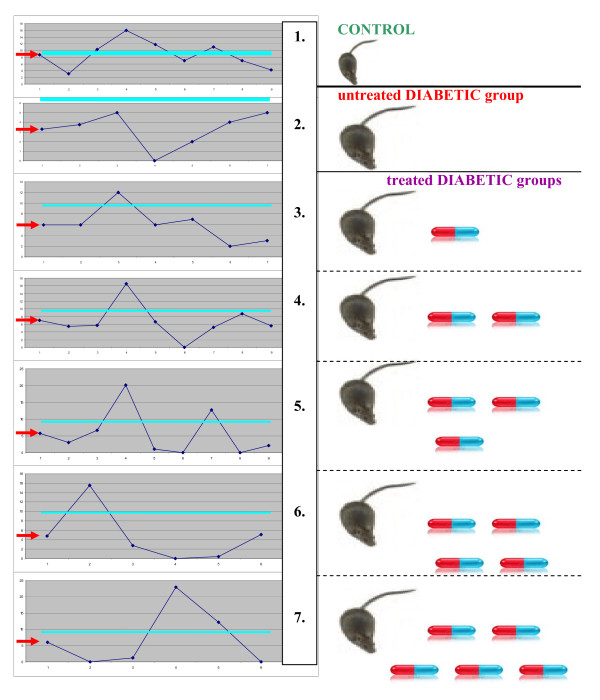
** Diagram demonstrates individual reactions towards CrPic treatments in the groups of comparison (1–7, group numbering is marked in the *middle*).** Individual levels of intact DNA (*axe X*, individual numbering; *axe Y*, comet class I in %) are highly heterogeneous in diabetic groups compared to control group 1. The highest level of heterogeneity is evident in groups 5, 6 and 7 with the highest doses of CrPic supplements (see ‘experimental design’ in Figure 3) The *turquoise lines* mark the level corresponding to the mean value of the control group; in contrast, the *red arrows* mark the level corresponding to the mean of the untreated diabetic group.

Subcellular imaging generally revealed group-specific patterns when diabetic animals were compared with controls, although higher heterogeneity was demonstrated within the untreated diabetic group: in some animals, intact DNA (class I comets) was monitored, whereas only comet classes with damaged DNA were monitored for others (see Figure 7). This heterogeneity clearly demonstrates an individualised reaction of organisms towards diabetic condition. This is a very important observation that corresponds well with the clinical picture of diabetes [8]. Hence, it is conclusive that the animal model used is suitable for issue-related studies to simulate the medical condition of diabetes and investigate individualised therapeutic effects. 

##### Highly individual reaction of diabetic animals towards CrPic dietary supplements

Despite group-specific patterns, the subcellular imaging by the comet assay indicated that each animal within a group responded individually to the identical dosage of CrPic administered and the treatment duration. Within the diabetic groups, experimental animals demonstrated highly individual comet patterns with respect to single dosages and treatment algorithms. This observation is important in regard of the highly individual response to the treatment algorithms applied. The paper concludes that there are individual reactions of diabetic animals towards doses and duration of CrPic treatment. This observation can explain discrepancies found in the literature concerning harmful effects of CrPic therapy [13,24-28].

The authors interpreted that CrPic treatment effects are unpredictable for the patient cohort as a whole, due to highly individual reactions towards therapy. Individuals should be treated personally on the basis of individual results going by predictive diagnostics and therapy monitoring.

##### Group-specific comparative quantification of apoptosis under CrPic treatments

**Figure 8:**
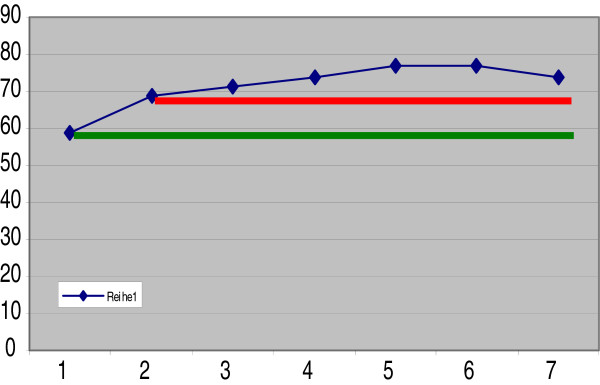
** Diagram presents mean values of individual experimental groups for the comets of class IV (apoptosis): *axe X*, group numbering (1–7); *axe Y*, corresponding mean values (% of comets).** The lowest rates correspond to control group 1 (*green line*); the highest rates correspond to diabetic groups treated with high doses of CrPic, namely experimental groups 5, 6 and 7. The *red line* marks the level corresponding to the mean value of the untreated diabetic group.

All diabetic groups demonstrate apoptotic rates which are significantly higher compared to the control group (see Figure 8). The highest rates of apoptosis were monitored in the diabetic groups treated with the highest doses of CrPic, namely in the groups 5, 6 and 7.

### Assumed and registered adverse health outcomes by CrPic treatments

Taken the above observations together, the authors conclude possible risks for individual long-term effects when CrPic is freely used as a therapeutic nutritional modality agent without application of advanced diagnostic tools to predict individual outcomes.

#### In experimental models

In terms of potential treatments of gestational diabetes, there is very little information about the safety of CrPic applied as a dietary supplement in pregnant women. However, experiments performed with CrPic administered to pregnant mice resulted in skeletal birth defects in the developing fetus [33].

Comparative cytotoxic and genotoxic studies of trivalent chromium demonstrated that the compound’s safety depends on the ligand bound to the chromium ion [34]. Thereby, CrPic produces significantly more oxidative stress and DNA damage compared, for example, to niacin-bound chromium(III). The implicated toxicity of CrPic may result in renal impairment; severe biochemical, histological and morphological changes in the eye; skin blisters and pustules; anaemia; hemolysis; tissue oedema; liver dysfunction; neuronal cell injury; impaired cognitive, perceptual and motor activity; enhanced production of hydroxyl radicals; chromosomal aberration; depletion of antioxidant enzymes; oxidative stress and DNA damage [23,29,34-38]. Increased apoptotic effects by CrPic were demonstrated by the authors of this study for kidney and for circulating leukocytes elsewhere [17,39]. Potential carcinogenic effects are assumed [40,41].

#### In humans

A growing body of case reports warns against adverse health outcomes assigned to CrPic dietary application, whereas by others, they are interpreted as ‘anecdotal reports’ [42]. Hence, case reports have described acute kidney failure, liver damage and anaemia by taking high dosage of CrPic as a dietary supplement [43,44]. Adverse cutaneous reactions to CrPic supplements have also been described [45].

Furthermore, there are some concerns that CrPic may affect the levels of neurotransmitters leading to potential risks for patients treated for depression, bipolar disorder and schizophrenia [18]. In a broader sense, CrPic supplements are hormone-related and may influence hormone secretion through their function in the endocrine/metabolic system [46]. Chromium supplements taken together with medications that block the formation of prostaglandins, such as ibuprofen, indomethacin, naproxen and aspirin, may increase the absorption of chromium in the body followed by unpredictable consequences such as long-term genotoxic effects caused by formation of covalent bonds to DNA molecules [26]. Some additive medication effects may be expected if CrPic therapy is combined with diabetes treatments, causing blood glucose levels to dip too low.

Here, the authors are wishing to stress the point that according to our analysis, the above listed adverse health effects are not expected to happen to everybody. They may occur individually with a severity grade depending on individual predispositions but can be effectively avoided by application of advanced diagnostic tools for the therapy monitoring and to predict individual outcomes. Further, the authors appeal to react effectively towards the case reports demonstrating adverse health outcomes by artificial dietary supplements often regarded as harmless by the public and lay media.

## Conclusions and recommendations

### Artificial supplements for diabetes prevention: hype or hope?

Definitely, CrPic therapy is solely one example of several therapy forms which currently are applied ‘across-the-board’ in diabetes care. The administration of artificial supplements is an attempt to prevent or at least to postpone the onset of type 2 diabetes in the group of risk and with individuals who are unwilling to make prudent changes in their diets and sedentary habits. Should this approach be considered as the hype or the hope? Considering type 2 diabetes as a multifactorial disease, our answer is that the above question is rather of rhetoric nature.

High efficacy of a balanced diet, an individually optimised lifestyle and personalised treatment regiments can hardly be substituted by a limited number of single supplements to cover all the multifactorial risks such as the upward trends of population ageing, environmental risk factors, urbanisation, additive effects of diverse stress factors, incorrectly chosen lifestyle including unfavourable nutritional habits, increasing prevalence of obesity, low physical activity, etc.

Although ageing is the well-acknowledged factor contributing to the disease’s development, there are completely new epidemiologic factors characteristic of the twenty-first century that speed up the disease’s progression particularly in the youth and in the young adults. Hence, it has been demonstrated that the prognosed DM rate progression will be inversely increasing with age (younger age = higher progression), and the youngest group of 20–39-year-old people will be delivering the highest rates of diabetic progression which will double the diabetes mellitus cohort of this age group by the year 2030 compared to 2010 [47]. This is a completely new situation and a very big challenge for most societies around the globe, requiring special competencies of several groups of professionals as well as innovative approaches in healthcare and health economy.

The population at-risk for diabetes is huge and increasing in a pandemic scale. One of the reasons might be the failed attempt to prevent the disease by the application of artificial supplements and drugs with hardly recognised individual risks. Consequently, a multimodal approach of integrative medicine by predictive diagnostics, targeted prevention and individually created treatment algorithms is highly desirable.

As discussed and reviewed earlier, more individualised treatments are desirable in effective protection against diabetic retinopathy and polyneuropathy, diabetes-related cardiovascular complications and cancer [8]. Further, field-related research is needed to establish simplified non-invasive diagnostic approaches for routine medical practice which would allow for an accurate prediction of individualised therapy risks and outcomes. A promising technological platform has been recently created using the detection of circulating nucleic acids in blood plasma [48] and clinical proteomics of body fluids [49].

Targeted measures require a creation of new guidelines that are essential to regulate (renoprotective) therapy approaches and the application of more individualised therapeutic modalities for advanced diabetes care. These measures should provide a legitimate regulation for well-timed predictive diagnostics, an effective prevention and the creation of individualised treatment algorithms in pre/diabetes [50].

## Competing interests

The authors declare that they have no competing interests.

## Authors’ contributions

KY participated in the design of the study and coordinated the performance of the experimental part. OG performed the design of the project and its coordination and created the concept of the manuscript. Both authors read and approved the final manuscript.
